# THE FEASIBILITY OF PERFORMING A VIRTUAL REALITY MEAL-PREPARING TASK IN YOUNG AND OLDER ADULTS

**DOI:** 10.1093/geroni/igae098.3557

**Published:** 2024-12-31

**Authors:** Oshadi Jayakody, Judy Pa, Joe Verghese, Hussain Doriwala, Robert Carrera, Sunil Agrawal, Helena Blumen

**Affiliations:** Albert Einstein College of Medicine, Bronx, New York, United States; University of California, San Diego, La Jolla, California, United States; Albert Einstein College of Medicine, Bronx, New York, United States; Columbia University, New York, New York, United States; Columbia University, New York, New York, United States; Columbia University, New York, New York, United States; Albert Einstein College of Medicine, Bronx, New York, United States

## Abstract

Virtual reality (VR) is an innovative technology to use in Alzheimer’s clinical trials to improve cognitive and behavioral skills in older adults. However, the application of VR in older adults raises questions regarding their ability to use VR platforms and concerns regarding simulator sickness (i.e., dizziness). We compared the task completion time, accuracy, simulator sickness and acceptability of a virtual meal-preparing task between 8 young (M age 25, SD 2.6, M years of education 17.1 SD 1.6, 50% women) and 8 older adults at risk for dementia (M age 76.3, SD 7.9, M years of education 12.9 SD 8.3, 62.5% women, 62.5% Black or Hispanic, screened with AD8 dementia screening). Following a practice task, all participants completed the meal-preparing task developed using Unity 3D (V2022 LTS). Participants were seated, wore a headset, and used wireless hand controllers to navigate and perform the task. Results from two-sample t-test showed that i) older adults took a longer time to complete the task (75 Vs 235 seconds; p< 0.001) and ii) made more errors (p=0.04) compared to young adults but no participants reported simulator sickness (i.e., dizziness, headache, eyestrain, etc.). All participants reported high self-efficacy in performing similar virtual tasks. This pilot study shows that, even though older adults required more guidance during the practice task, they were still able to complete a simple virtual task within an average of < 5 minutes. Additionally, they reported high acceptability (i.e., self-efficacy, level of enjoyment) and willingness to engage in future VR interventions.

